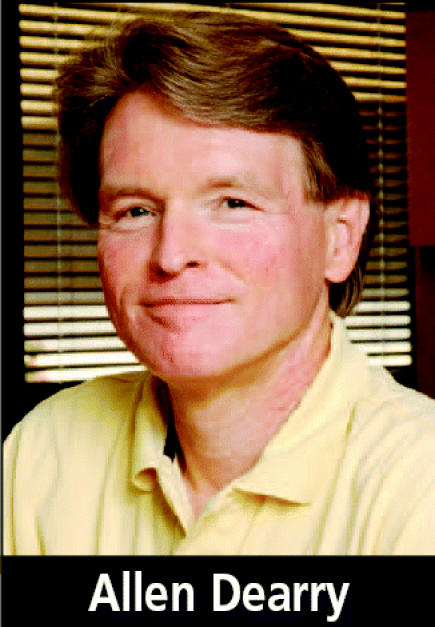# Editorial: Impacts of Our Built Environment on Public Health

**DOI:** 10.1289/ehp.112-a600

**Published:** 2004-08

**Authors:** Allen Dearry

**Affiliations:** NIEHS, National Institutes of Health, Department of Health and Human Services, Research Triangle Park, North Carolina, E-mail: dearry@niehs.nih.gov

We spend more than 90% of our lives indoors (National Research Council 1981), yet we know much more about ambient environmental factors and health than we do about the “built environment” and health. Conceptually, the built environment includes all of the physical structures engineered and built by people—the places where we live, work, and play. These edifices include our homes, work-places, schools, parks, and transit arrangements. How we design and build where we live has changed dramatically over the past century. In the early 1900s, urban areas tended to be compact and communities were walkable, with a central business district and a mix of housing and services. Then, connections between urban design and health and disease were more clearly recognized, and planners and public health practitioners often worked together to deal with problems related to poor sanitation and housing conditions. Increasing movement away from such urban locales over the last 50 years led to lower-density developments, segregation of land uses, and extensive roadway construction. Today, this trend, sometimes referred to as “urban sprawl,” is characterized by huge increases in urbanized land area and vehicle miles traveled [U.S. Environmental Protection Agency (EPA) 2001a]. These changes have both direct and indirect impacts on our environment and on public health.

Changes in land use and development patterns have contributed to habitat loss and declining water resources and quality (Soule 1991; U.S. EPA 1992). Increases in impervious surfaces and attendant surface water runoff contribute to deterioration in availability and use of safe, clean water supplies for both recreation and consumption. For example, suburban development is associated with a rising load of polycyclic aromatic hydrocarbons in nearby surface water (Van Metre et al. 2000).

Increases in vehicle travel affect our environment and our health in multiple fashions. As neighborhood density decreases, vehicle miles traveled (VMT) increase (Holtzclaw et al. 2002). With more driving comes more vehicle crashes as well as pedestrian injuries and fatalities. Moreover, further VMT contribute to overall releases of air pollutants (Kennedy and Bates 1989), which are associated with numerous adverse health outcomes (Samet et al. 2000). Additionally, carbon dioxide and other vehicle emissions contribute to accumulation of greenhouse gases in the atmosphere (U.S. EPA 2001b), which may ultimately impact public health by affecting the transmission and spread of infectious diseases (Epstein 2000).

Our built environment also affects individual mental health as well as population-wide well-being. Housing type and quality, neighborhood quality, noise, crowding, indoor air quality, and light have all been linked to personal mental health (Evans 2003). Indirectly, the built environment may influence development and maintenance of socially supportive networks within a community. Higher levels of this type of “social capital” are associated with lower levels of morbidity and mortality (Kawachi et al. 1999). Although the connection between the built environment and social capital remains to be well established, both walkability and mixed use of neighborhoods have been reported to be related to an enhanced sense of community and social capital (Glynn 1981; Nasar and Julian 1995).

Perhaps the most recently publicized link between the built environment and public health relates to the occurrence of overweight and obesity in the United States. The built environment influences weight management by affecting both food intake and energy expenditure. Communities characterized by less-dense development are associated with more vehicle travel and less walking and biking than are more densely developed communities (Frank and Pivo 1995). Physical activity has been shown to have a salubrious effect on health and quality of life (Lee and Paffenbarger 2000). However, only recently have investigators expanded such work to address more specifically the impact of community design not only on physical activity but also on obesity and associated comorbidities. One study reported that, after controlling for individual differences, those living in sprawling counties are more likely to walk less in their leisure time, weigh more, and have a greater prevalence of hypertension than those living in more compact places (Ewing et al. 2003). Similarly, a more walkable environment has been found to be associated with higher physical activity and lower obesity levels (Salens et al. 2003). In addition, the likelihood of obesity apparently declines with increases in mixed land use, but rises with increases in time spent in a car per day (Frank et al. 2004). To date, such work addresses important relationships but does not establish causation. In fact, Frank et al. (2004) pointed out that mixed land use, while being the most important variable of the built environment related to obesity, may not exert its effect via physical activity. Hence, significant methodologic and etiologic research remains to be conducted to clarify such issues.

The built environment may also play a role in controlling weight by shaping food access and availability. Recent research suggests that supermarkets are more likely to be located in wealthier and predominantly white areas, and that fruit and vegetable intake is positively associated with the presence of a supermarket, even after controlling for personal socioeconomic factors (Morland et al. 2002a, 2002b). Although the relationship between different types of eating places and dietary consumption has not been well examined, the availability, type, and distribution of restaurants and the diffusion of food advertising represent other means by which the environment may affect weight homeostasis.

Additional research will be necessary to enable us to understand the complicated pathways and intersections linking community design, transportation, and a variety of health outcomes. Such information will permit us to develop communities that promote health for both people and ecosystems rather than dealing with the health-damaging repercussions of a poorly designed built environment (Srinivasan et al. 2003). In pursuit of this goal, it will be important to reestablish the unity of health practitioners and public planners—not only to carry out needed research at the interface of these disciplines but also to ensure that the results of such research are properly translated and applied in order to lead to tangible improvements in our living arrangements and in public health.

## Figures and Tables

**Figure:**